# Does Language Proficiency Affect Completion of Telerehabilitation Video Visits? A Retrospective Study

**DOI:** 10.1007/s10903-025-01830-7

**Published:** 2025-12-01

**Authors:** Mansha Mirza, Hajwa Kim, Courtney Pilat

**Affiliations:** 1 Present Address: University of Illinois Chicago, Chicago, USA; 2 OSF HealthCare, Peoria, USA

## Abstract

Telehealth use increased dramatically after the COVID-19 pandemic enabled largely by regulatory changes. Health policy experts recommend telehealth as a viable option for healthcare delivery in the post-pandemic world. As investment in telehealth continues, it is important to ensure that existing health disparities are not exacerbated. One group that is particularly vulnerable to telehealth challenges includes patients with limited English Proficiency. This study aimed to examine whether English proficiency is associated with completion of telerehabilitation visits. Electronic Health Records were retrieved for all patients scheduled for a telerehabilitation visit (physical/occupational therapy) in adult or pediatric outpatient clinics at a large academic medical center between April 1, 2020 and April 30, 2022. Using data from 3,871 video visits involving 1414 patients, binomial logistic regression models were estimated to examine associations between sociodemographic variables (age, gender, race, ethnicity, insurance status, income, language preference) and visit completion rate using multivariable logistic regression models. 6.4% of telerehabilitation visits were conducted in a language other than English. 24% patients had a 0% completion rate, 9% had a completion rate of > 0% and < 100%, and 67% had a 100% completion rate. Being older and publicly insured was significantly associated with decreased odds of 100% completion. Patients with English as their preferred language showed higher odds of visit completion. Age, type of insurance, and language preference were associated with successful completion of telerehabilitation video visits. Future research is needed to reveal specific barriers to telerehabilitation for older patients and those with limited English proficiency.

**Supplementary Information:**

The online version contains supplementary material available at 10.1007/s10903-025-01830-7.

## Background

Telehealth use in the US increased dramatically during the COVID-19 pandemic enabled largely by regulatory changes that expanded public and private insurance coverage for video- and phone-based delivery of healthcare services [1]. Although telehealth utilization has dropped since the peak of the pandemic, usage and patient acceptance remains higher than pre pandemic levels as indicated by national trends [2] as well as data from specific health systems [3] and geographic communities [4]. Available evidence also allays some concerns about telehealth potentially duplicating in-person health visits. Research shows that telehealth substitutes for rather than duplicates certain types of in-person visits such as consultations for common acute outpatient conditions [5], primary care visits [3, 6] especially when the patient has an existing clinical relationship with the provider [7], and visits with specialists that do not require in-person hands-on care such as genetics, nutrition, mental health, diabetes care [8]. Additionally, analysis of federal claims data and electronic health records from large, integrated health system in the US suggests that telehealth does not appear to increase downstream emergency department visits [9, 10], and does not trigger non-essential return visits or follow ups across primary and specialty care [8, 9]. Studies have also found telehealth to be clinically useful and cost effective in chronic disease management [10, 11] and for improving access to continuity of behavioral health services [12].

The continuing utility and promise of telehealth have prompted bipartisan legislation to incrementally extend pandemic telehealth provisions beyond the initially approved timeline. Notably, professional bodies such as the American Medical Association and the American Hospital Association strongly support permanent expansion of telehealth access [13, 14]. With some pandemic telehealth provisions becoming permanent and others being extended, telehealth is emerging as a viable option for healthcare delivery in the post-pandemic world [15]. As we develop systems and practices to routinize telehealth, it is important to ensure that existing health disparities are not exacerbated.

One group that is particularly vulnerable to telehealth challenges includes patients with Limited English Proficiency (LEP). This group includes individuals whose primary language is not English and who have limited ability to read, write, speak, and understand English [16]. In 2021, 25.7 million individuals in the US were deemed to have LEP, comprising 8% of the overall population aged five years and older [17]. Despite federal regulations mandating equitable healthcare access for patients with LEP, this group experiences significant challenges even with conventional in-person healthcare [18, 19]. Communication barriers in the absence of bilingual providers and professional interpreters contribute to high rates of misdiagnosis, over or under treatment, adverse events, and treatment-related complications, resulting in poor health outcomes and low patient satisfaction [19–21].

Disparate telehealth utilization for patients with LEP has been documented well before the telehealth surge during the COVID-19 pandemic. In a representative sample of California adults from 2015 to 2018, patients with LEP had half the odds of using telehealth services compared with English-proficient patients, even after accounting for sociodemographic characteristics, health status, and internet use [22]. Similar disparities were noted during the COVID-19 pandemic with multiple studies reporting LEP to be independently and significantly associated with lower telehealth use [23, 24]. One study found that primary care visits for patients with LEP decreased significantly after the pandemic pivot to telehealth [25], while another found that telehealth visits for specialty care were less likely to be completed for patients whose preferred language was not English [26].

While studies have shed light on telehealth disparities for patients with LEP in primary care and other medical specialties, little is known about telehealth inequities in the context of rehabilitation services, such as physical and occupational therapy. Available research on in-person encounters has identified unique communication challenges distinct from other healthcare services during rehabilitation encounters with patients with LEP [27, 28]. Therefore, research on telehealth in the context of other healthcare settings might not directly translate to rehabilitation services. This paper stems from a larger concurrent mixed methods study that sought to investigate disparities in outpatient telerehabilitation delivery for patients with Limited English Proficiency (LEP) as well as to identify barriers to and facilitators of successful telerehabilitation for this population. In this paper, we present procedures and findings related to the first aim which was to examine whether English proficiency is associated with completion of telerehabilitation video visits. We hypothesized that LEP would be independently associated with fewer completed telerehabilitation video visits (as opposed to cancelled visits or no shows).

## Conceptual Framework

The larger mixed methods study drew upon the National Institute on Minority Health and Health Disparities Research Framework Expanded for Digital Health Equity [29]. The framework includes four levels of influence (individual, interpersonal, community, societal) and six domains of influence (biological, behavioral, physical environment, digital environment, sociocultural environment, and health care system). For this study, we considered completion of rehabilitation visits as an individual health outcome with LEP as an explanatory variable within the domain of sociocultural environment. We also included demographic factors and other covariates within the sociocultural environment and health system domains. For the concurrent qualitative aim of the larger study (not presented here), we also considered aspects of the digital environment at the individual, interpersonal, and community levels.

## Methods

Electronic Health Records (EHRs) were retrieved for all patients scheduled for a telerehabilitation visit (physical/occupational therapy) in adult or pediatric outpatient clinics at a large academic medical center between April 1st 2020 and April 30th 2022. This date range was selected as it represents the first two years of the COVID-19 pandemic. During the first year, most outpatient rehabilitation visits were taking place via telehealth. During the second year, rehabilitation clinics were gradually opened for in-person visits.

The outcome variable was completion status of a telerehabilitation visit as indicated in the encounter’s electronic record (‘completed’ versus ‘cancelled’, ‘no show’, ‘left without being seen’, ‘arrived, not completed’). In the EHR data warehouse, no telephone encounters were associated with occupational/physical therapy visits. All telehealth encounters for these visits were labelled as ‘video’ encounters. Therefore, the dataset used in this study only includes visits scheduled to be held via video conference. Phone visits were not included.

Visits were conducted either by making a secure video call using an external telehealth app or by using the EHR system’s in-built video visit functionality. When needed, interpreters were integrated into the video call. Neither the app nor the EHR system included language localization features. Patients with LEP were not provided any additional supports or instructions for joining the video visit.

The main independent variable was LEP and was defined based on preferred spoken language listed in the EHR. If a language other than English was listed, the encounter was classified as involving a patient with LEP. Covariates included self-identified demographic characteristics such as age, gender, race, and ethnicity, health insurance (public versus private insurance coverage) and socioeconomic status. Due to large amounts of missing data for income and education level, we created a proxy measure of patient socioeconomic status. We used median household income data from the American Community Survey (2022 inflation-adjusted dollars) matched to each patient’s zip code. Income levels were subsequently classified as low (<$45,000), middle (>=$45,000 and < 131,000) and high (>=$131,000) based on recommendations for households in the metropolitan catchment area of the academic medical center [30].

This study was reviewed and approved by the Institutional Review Board at the lead author’s academic institution, and a waiver of informed consent was granted as the present study focused on analyzing deidentified secondary data.

Statistical Analyses:

The outcome was defined as visit completion rate and was calculated as the total number of completed visits of all scheduled telerehabilitation visits within each patient. The distribution of completion rate was categorized into three groups, 0%, greater than 0 to less than 100%, and 100%. The association between independent variables and the outcome was examined in bi-variate analyses using chi-square tests for categorical variables and ANOVA tests for continuous variables. Multivariable logistic regression models were used to test the effects of language preference on visit completion rate along with other covariates including age and insurance status. All analyses were performed using SAS 9.4 and statistical significance level of *p* < 0.05.

## Results

Data were analyzed from 3,871 visits involving 1414 patients; 6.4% of telerehabilitation visits were conducted in a language other than English. Table 1 summarizes patient demographic data. Of the 1,414 patients with scheduled telerehabilitation visits, 340 (24%) had a 0% completion rate, 133 (9%) had a completion rate of > 0% and < 100%, and 942 (67%) had a 100% completion rate.

**Table 1:** Demographic characteristics of sample

Characteristics	Total (*n* = 1414)
	**Mean (SD, IQR)**
**Age**	37.3 (20.6, 24–53)
	**n (%)**
**Gender**	
Female	987 (69.9)
Male	425 (30.1)
**Ethnicity**	
Hispanic	324 (23.9)
Non-Hispanic	1029 (72.8)
**Race**	
Asian	50 (3.5)
Black	744 (52.6)
Hawaiian	1 (0.1)
Native American	6 (0.4)
White	236 (16.7)
Not indicated	377 (26.7)
**Insurance status**	
Public health insurance	737 (68.5)
Private health insurance	330 (30.7)
Self-pay	9 (0.8)
**Neighborhood Median Income**	
Low	305 (21.6)
Middle	1072 (75.8)
High	33 (2.3)

Bivariate analyses (Supplementary Table 1) showed that age and insurance type were the only independent variables that were significantly associated with visit completion rate. Bivariate analyses also showed that preferred language was associated with race and ethnicity variables. Therefore, the latter variables were not included in the final multivariable model.

In multivariate analyses, age (*p* < 0.01) and insurance type (*p* = 0.03) were significantly associated with visit completion rate. Specifically, older age and being publicly insured were associated with decreased odds of having 100% completion rate for telerehabilitation visits. Language preference had a marginally significant association with visit completion rate (*p* = 0.05). Patients with English as their preferred language showed higher odds of having either a 100% or 0 to100% completion rate over a 0% completion rate for telerehabilitation visits. See Table 2 for details.

**Table 2:** Multivariable logistic regression model

Variable	Odds Ratio Estimate	95% Wald Confidence Limits		*p*-value
**Age (continuous)** > 0% and < 100% 100%	0.990 0.977	0.972 0.963	1.009 0.992	0.30 0.00
**Gender (Female vs. Male)** > 0% and < 100% 100%	1.404 1.120	0.601 0.566	3.282 2.214	0.43 0.75
**Language Preference (English vs. Other)** > 0% and < 100% 100%	4.481 2.650	1.089 1.109	18.441 6.333	0.04 0.03
**Insurance Coverage (Private vs. Public)** > 0% and < 100% 100%	2.429 2.989	0.951 1.318	6.203 6.780	0.06 0.01
**Neighborhood Median Income (Low vs. High)** > 0% and < 100% 100%	0.735 0.951	0.059 0.116	9.200 7.801	0.55 0.62
**Neighborhood Median Income (Middle vs. High)** > 0% and < 100% 100%	1.291 1.620	0.110 0.205	15.170 12.800	0.55 0.37

## Discussion

Overall, findings of this study are consistent with previous research investigating characteristics associated with telehealth access and utilization. Older patients, those with a non-English language preference, and those with public insurance were more likely to have incomplete video visits for rehabilitation care. Previous studies have documented similar disparities for video telehealth related to older age, language barriers, and reliance on public insurance in a variety of healthcare settings such as adult primary care, internal medicine, geriatric care, cancer care, pulmonary care, infectious disease care, cardiology, rheumatology, gastroenterology, and nephrology [26, 31–34]. This study adds to this body of literature, being the first to focus on telehealth disparities in the context of rehabilitation care provided via video conference.

A unique aspect of rehabilitation care is that patients typically need multiple visits conducted over a regular schedule (e.g. weekly) and within a designated timeframe for optimal recovery. In the post-pandemic world, these visits can be a mix of in-person and telerehabilitation depending on patient needs and proximity to rehabilitation services [35]. This mixed visit structure offers opportunities to support video visit uptake among patients who might be susceptible to telehealth disparities. For example, previous literature on telehealth with LEP populations suggests that when patients arrive for in-person visits, patient navigators and bilingual community health workers can provide them with telehealth literacy training [36, 37]. Support staff can be stationed in clinic waiting rooms to help patients enroll in the patient portal if this is a required step for participation in video visits [38, 39]. Prior studies have shown that this type of patient education can increase the likelihood of successful video visits [34].

Another strategy recommended in the literature that has shown preliminary efficacy for improving the success of video visits specifically for patients with LEP is virtual rooming. This involves a medical assistant conducting a pre-visit check with the patient to troubleshoot technology and prepare for the visit [40]. For video telerehabilitation visits, this check in may be conducted by a patient navigator or other support staff and should include customized visit preparation such as keeping adaptive aids ready and tips for camera positioning so the provider can easily assess limb function, balance, reach and other rehabilitation outcomes. This type of support might not be needed indefinitely. Existing evidence suggests that for patients with LEP, prior success with video visits is associated with higher video visit frequency in the future [23]. Thus, handholding patients through the first video visit will likely be sufficient for independently completing video visits in the future.

Provider trainings can also contribute to greater uptake and success of video telerehabilitation visits. Such trainings might include continuing education courses that combine telehealth competencies with cross-cultural communication skills [41], discipline-specific case studies, and simulation-based learning experiences [42] that are customized for rehabilitation professionals. Broader changes within healthcare systems are also needed such as providing end-to-end language supports i.e. from the time of scheduling an appointment to post-visit follow up. Healthcare systems should also conduct formal outreach to patients with LEP and provide telehealth setup instructions in non-English languages that are most commonly spoken among their patient population [38].

## Limitations

Our study had a few limitations. We used EHR data from a single academic center located in a large metropolitan city which might not be representative of the larger US population. The EHR records retrieved also had some limitations such as missing data for socioeconomic variables. The EHR database available for this study also did not include scheduling information for rehabilitation visits that might have been conducted by phone during the study period. Therefore, we were unable to examine completion rates for telephone-based, voice-only telerehabilitation visits. We used language preference as a proxy measure for language proficiency. Although measures of language proficiency are considered more robust, prior research has found proficiency measures and preference measures to be moderately to highly correlated [43]. The use of retrospective quantitative data did not allow us to investigate specific barriers to telerehabilitation such as digital literacy, access to technology, cultural preferences for in-person contact, and physical/sensory impairments – the latter being more common among users of rehabilitation services. Future qualitative research is needed to shed light on these factors.

## Conclusions

This retrospective analysis of electronic health records identified patient characteristics associated with successful completion of telerehabilitation visits with adult or pediatric outpatient clinics at a large academic medical center. Older age, being publicly insured, and preference for a non-English language were associated with decreased odds of having 100% completion rate for telerehabilitation visits. These findings are consistent with previous research on telehealth utilization in other healthcare settings. Future research is needed to reveal specific barriers to telerehabilitation for patients with these characteristics and to identify best practices for addressing these barriers.

## Supplementary Information

Below is the link to the electronic supplementary material.

Supplementary Material 1

## Data Availability

The data that support the findings of this study are not openly available due to reasons of sensitivity and are available from the corresponding author upon reasonable request, and with permission from the Clinical Research Data Warehouse at the University of Illinois Chicago.
