# Supplementary material for: Does Language Proficiency Affect Completion of Telerehabilitation Video Visits? A Retrospective Study
Source: J Immigr Minor Health. 2025 Dec 1;28(4):863–9. doi: 10.1007/s10903-025-01830-7 (PMC13451519; doi:10.1007/s10903-025-01830-7)
Supplement: Supplementary file 1 [file 10903_2025_1830_MOESM1_ESM.pdf]

Supplementary Table 1. Bivariate Analyses

Independent Variables	Visit Completion Rate			
	**0%<br>n (percent)**	**> 0% and < 100%<br>n (percent)**	**100%<br>n (percent)**	χ^2
**Gender**				
Male	101 (24)	34 (8)	290 (68)	1.
Female	239 (24)	99 (10)	650 (66)	
**Race**				
Black	186 (25)	63 (8)	496 (67)	3.
Asian	12 (24)	4 (8)	34 (68)	
White	55 (23)	27 (11)	154 (65)	
Native American or Hawaiian	3 (43)	0 (0)	4 (57)	
**Ethnicity**				
Hispanic	73 (22.5)	29 (9)	222 (68.5)	0.
Non Hispanic	255 (25)	93 (9)	682 (66)	
**Language Preference**				
English	306 (24)	123 (9)	876 (67)	1.
Other	26 (29)	8 (9)	56 (62)	
**Insurance Coverage**				
Public	47 (6)	60 (8)	630 (86)	9.
Private	7 (2)	25 (7)	308 (91)	
**Neighborhood Median Income**				
Low	85 (28)	28 (9)	192 (63)	4.
Middle	242 (22)	103 (10)	728 (68)	
High	10 (30)	2 (6)	21 (64)	
	**Mean (sd)**	**Mean (sd)**	**Mean (sd)**	**F**
**Age**	42.93 (19.02)	34.85 (20.88)	40.12 (18.90)	2

*There were missing data for all independent variables due to participant non-response. The data presente
frequencies and percentages.
